# Enhanced Anticancer Activity of Gemcitabine in Combination with Noscapine via Antiangiogenic and Apoptotic Pathway against Non-Small Cell Lung Cancer

**DOI:** 10.1371/journal.pone.0027394

**Published:** 2011-11-15

**Authors:** Mahavir B. Chougule, Apurva Patel, Pratik Sachdeva, Tanise Jackson, Mandip Singh

**Affiliations:** 1 Department of Pharmaceutical Sciences, College of Pharmacy, University of Hawaii, Hilo, Hawaii, United States of America; 2 Department of Pharmaceutical Sciences, College of Pharmacy and Pharmaceutical Sciences, Florida A&M University, Tallahassee, Florida, United States of America; The University of Kansas Medical Center, United States of America

## Abstract

**Background:**

The aim of this investigation was to evaluate the anticancer activity of Noscapine (Nos) and Gemcitabine (Gem) combination (NGC) against non-small cell lung cancer (NSCLC) and to elucidate the underlying mechanism of action.

**Methods:**

Isobolographic method was used to calculate combination index values from cytotoxicity data. In vitro antiangiogenic and apoptotic activity of Nos, Gem and NGC was evaluated. For in vivo studies, female athymic Nu/nu mice were xenografted with H460 tumors and the efficacy of Nos, Gem, or NGC was determined. Protein expressions by immunohistochemical staining were evaluated in harvested tumor tissues.

**Results:**

The CI values (<0.59) were suggestive of synergistic behavior between Nos and Gem. NGC treatment showed significantly inhibited tube formation and increased percentage of apoptotic cells. NGC, Gem and Nos treatment reduced tumor volume by 82.9±4.5 percent, 39.4±5.8 percent and 34.2±5.7 percent respectively. Specifically, NGC treatment decreased expression cell survival proteins; VEGF, CD31 staining and microvessel density and enhanced DNA fragmentation and cleaved caspase 3 levels compared to single agent treated and control groups.

**Conclusion:**

Nos potentiated the anticancer activity of Gem in an additive to synergistic manner against lung cancer via antiangiogenic and apoptotic pathways. These findings suggest potential benefit for use of NGC chemotherapy for treatment of lung cancer.

## Introduction

Lung cancer is the leading cause of cancer-related deaths. Majority of patients diagnosed with lung cancer present with locally advanced or metastatic disease [1], [2]. More than 85 percent of patients with lung cancer have non-small cell lung cancer (NSCLC). Majority of newly diagnosed NSCLC patients present with disease beyond the scope of surgical cure and depend on systemic chemotherapy to improve their outcome [3], [4]. Platinum based combination regimens are first-line treatment option in treatment of NSCLC but their clinical utility has been limited due to substantial toxicities [4], [5]. Despite recent advances in chemotherapy, response rates in NSCLC remain <50 percent and a third of patients with stage IV disease have a 2-year survival rate of <20 percent [3]. The establishment of an optimal regimen for combination therapies with currently used and newly developed drugs is an important step to achieve higher response and longer survival [6]. To address this problem, attention has been focused on finding novel anticancer agents that will deliver equivalent or improved survival to that achieved with platinum regimens, but with less toxicity. Gemcitabine (Gem) is a pyrimidine nucleoside antimetabolite agent which is active against variety of human malignancies, including NSCLC with a favorable toxicity profile [6], [7]. Several researchers have studied the combination of Gem and cisplatin, topotecan, protease inhibitors, and ginsenoside Rg3 for the treatment of lung cancer [8], [9], [10], [11], [12]and reported enhanced anticancer effects [8], [9], [10], [11], [12], [13].

The anticancer activity of microtubule-interfering agents, taxanes and vinca alkaloids has been well studied [14]. However, the clinical utility of taxanes has been limited due to drug-resistance, need of intravenous (i.v.) infusion over a long period of time and associated toxicities [15], [16]. This has prompted search for microtubule-targeting agent that may be administered orally, display favorable toxicity profiles and have better therapeutic indices. Nos attenuates microtubule dynamics just enough to activate the mitotic checkpoints to stop cell cycle [17], [18] and demonstrated anti-proliferative activity against wide variety of cancer cells including many drug-resistant variants while evading normal cells [18], [19], [20], [21], [22], [23], [24], [25], [26]. Furthermore, Nos also showed little or no toxicity to the normal organs and did not inhibit primary humoral immune responses in mice [19], [20]. Our previous studies demonstrated that oral administration of Nos showed significant anticancer activity in a dose-dependent manner against H460 lung tumor xenografts [24]. Landen et al. demonstrated that there was no significant improvement in the anticancer activity of Nos when combined with paclitaxel[19] possibly due to competition for the same target. The use of Nos in combination with vincristine exhibits synergistic antitumor effects in leukemia cells in vitro [27]. However, anticancer potential of Nos in combination with various anticancer agents in the treatment of lung cancer has not been systematically explored. Both Nos and Gem have different mechanism of action and Nos and Gem combination (NGC) may lead to potential synergistic antitumor activity against lung cancer.

Based on the individual activity of these agents and their distinct mechanisms of action, we hypothesized that NGC may produce additive to synergistic cytotoxic effects in human lung cancer cells *in vitro* and *in vivo* possibly by degradation of specificity proteins, enhancing antiangiogenic and apoptotic activity. In present investigation, we evaluated anticancer activity of NGC therapy against NSCLC cells in vitro and in vivo in H460 murine xenograft lung tumor model which has not been reported before. The objectives of this study were to (a) examine the anticancer activity of combination of between Nos and Gem against NSCLC cells, and (b) evaluate the antitumor effect of NGC in mice bearing H460 xenograft lung tumors and elucidate underlying mechanism of action.

## Materials and Methods

### Materials

Noscapine and Gemcitabine were purchased from Sigma Chemicals, St. Louis, MO, USA and Spectrum Chemicals USA respectively. The human H460 and A549 NSCLC cells were obtained from American Type Culture Collection (Rockville, MD, USA). All other chemicals were either reagent or tissue culture grade. H460 and A549 cells were grown in RPMI 1640 medium and F12K medium (Sigma, St. Louis, MO, USA) supplemented with 10 percent fetal bovine serum respectively. All tissue culture media contained antibiotic antimycotic solution of penicillin (5000 U/ml), streptomycin (0.1 mg/ml), and neomycin (0.2 mg/ml). The cells were maintained at 37°C in the presence of 5 percent CO_2_ in air.

### Animals

Female Nu/Nu mice (six weeks old, Harlan, Indianapolis, IN) were grouped and housed (8/cage) in sterile microisolator caging unit supplied with autoclaved Tek-Fresh bedding. The animals were housed at Florida A and M University in accordance with the standards of *the Guide for the Care and Use of Laboratory Animals* and the Association for Assessment and Accreditation of Laboratory Animal Care. The present study was reviewed and approved by Florida A and M University (FAMU) Animal Care and Use Committee (AUCU) August, 2009. (Protocol # 002-09).

### *In vitro* cytotoxicity studies

The A549 or H460 cells were plated in 96-well micro titer plates, at a density of 1×10^4^ cells/well and allowed to incubate overnight. The cells were treated with various dilutions of Gem in the presence or absence of Nos at 10–30 and 30–50 µM against H460 and A549 cells respectively. The plates were incubated for 72 h at 37±0.2°C in a incubator. Cell viability in each treatment group was determined by crystal violet dye assay.

### Induction of apoptosis in H460 and A549 cells

The H460 or A549 cells were plated at a density of 1×10^6^ cells/well in 6-well plates and incubated overnight. H460 cells were treated with Gem (0.4 µg/ml), or Nos (30 µM), or NGC and A549 cells were treated with Gem (0.3 µg/ml), or Nos (50 µM), or NGC. After 72 h, cells were fixed in 4% paraformaldehyde and mounted onto slides using Cytospin R (Shandon) and processed as per ApoTag Red In Situ Apoptosis detection kit R (Chemicon R International, CA, USA) protocol. The images on the slides were visualized with an Olympus BX40 fluorescent microscope. To quantify the apoptotic cells, 100 cells from 6 random microscopic fields were counted.

### Inhibition of tube formation of HUVEC cells *in vitro*

The antiangiogenic effects of Nos and Gem were analyzed using an *In Vitro* Angiogenesis Assay Kit (Millipore, Billerica, MA, USA). Briefly, HUVEC cells were cultured in the presence or absence of 30 µM Nos, 0.4 µg/ml Gem and NGC on polymerized Matrigel at 37°C. Standard Matrigel was allowed to polymerize in a 96-well plate and HUVEC cells were seeded at a density of 3×10^4^ per well in ECM medium. After 6 h, tube formation by endothelial cells was evaluated and photographed. Quantification of progression of angiogenesis was accomplished by counting the capillary tube branch points formed after a set amount of time (end-point assay). Branch points in several random view-fields (3–10) per well should be counted and the values averaged.

### *In-vivo* antitumor effect of Nos against H460 lung tumors

The adherent H460 cells were harvested and centrifuged at 500 g for 4 min at 4°C and the cell pellet was resuspended. The cells were diluted to 3×10^6^ cells/100 µl using growth medium. The 100 µl of cell suspension was injected subcutaneously into right flank area of each mouse. The protocol for *in-vivo* experiments with nude mice was approved by the Animal Care and Use Committee, Florida A and M University, Tallahassee, FL. The mice were randomized (n = 8) after 50 mm^3^ of tumor xenografts (7 days post tumor implantation) and treated with i) 160 µl of vehicle (Phosphate buffer, pH 3.5); ii) Gem (30 mg/kg i.v. bolus, q3d ×7 schedule); iii) Nos 300 mg/kg daily by oral gavage; and iv) NGC therapy for 38 days post tumor implantation_._ Noscapine was dissolved in phosphate buffer, pH 3.5 and Gemcitabine was dissolved in the saline phosphate buffer before administration to mice. To check for evidence of toxicity, the animals were weighed twice weekly. The tumor dimensions were measured using a linear caliper and tumor volume was calculated using following equation:
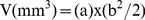
(1)where,

v = tumor volume

a = largest diameter of tumor

b =  smallest diameter of tumor

On day 38, all animals were sacrificed following removal of the tumor tissues; some of the tumors were fixed in formalin while others were rapidly frozen in liquid nitrogen and stored in −80°C.

### Western Blotting of Tumor Tissues

The proteins were extracted from tumor tissue using RIPA buffer with protease inhibitor incubated for 30 min on ice and the supernatants were stored at –80°C after centrifugation. For western blotting (WB), a previously established procedure in the laboratory was used. [24], [28] The membranes were probed with primary antibodies (Cell Signaling Technology, Beverly, MA) Sp1 (1∶750), Sp3 (1∶750), VEGF (1∶500), pAKT (1∶500), Cyclin D1(1∶500), p53 (1∶500), p21 (1∶500),), PARP (1∶1000), cleaved PARP (1∶1000), cleaved caspase 3 (1∶1000), caspase 8 (1∶1000) and caspase 9 (1∶1000), Bax (1∶1000), Bcl_2_ (1∶1000), BID (1∶500) and β-actin antibodies (1∶500). Bound antibodies were revealed with HRP conjugated secondary antibodies (1∶2000) using SuperSignal West pico chemiluminescent solution (Pierce, Rockford, IL). Beta actin protein was used as a loading control. The densitometric analysis of the bands was performed using the program ImageJ v1.33u.

### TUNEL Assay, Cleaved Caspase 3 expression and VEGFexpression

The TUNEL, cleaved caspase-3 and VEGF staining in paraffin-embedded tumor tissues sections were evaluated using DeadEndTM Colorimetric Apoptosis Detection System (Promega, Madison, WI), cleaved caspase-3 staining kit (Cell Signaling Technology, Beverly, MA) and ImmunoCruz™ ABC Staining System (Santa Cruz Biotechnology, Inc., Santa Cruz, CA) respectively as described previously [26]. Tumor tissue sections (4–5 µm thick) mounted on poly-L-lysine–coated slide were deparaffinized by xylene and dehydrated through graded concentrations of alcohol, then incubated with 3 percent hydrogen peroxidase for 20 min to block endogenous peroxidase activity. Antigen retrieval for staining and blocking of endogenous peroxidase was performed as described previously [26]. The samples were incubated overnight at 4°C with 1∶50 dilution of primary antibody incubated with biotinylated secondary antibody followed by streptavidin. The color was developed by exposing the peroxidase to a substrate-chromagen, which forms a brown reaction product. The sections were then counterstained with hematoxylin. Three slides per group were stained and TUNLE, cleaved caspase 3 stained cells were identified by dark brown cytoplasmic staining. The VEGF stained cells were identified by brown staining. The images on the slides were visualized with an Olympus BX40 light microscope.

### CD31 Expression and Assessment of Microvessel Density

For CD31 expression after washing with PBS, the sections were pretreated in citrate buffer in a microwave oven for 20 min at 92–98°C. After two washes with PBS, specimens were incubated in 10 percent normal goat serum (Atlanta Biologicals, GA, USA) for 20 min to reduce the nonspecific antibody binding. Subsequently, the sections were then incubated with a 1∶500 diluted mouse CD31 monoclonal antibody (Cell Signaling Tech, MA), which is recognized as an endothelial cell surface marker, at room temperature for 1 h, followed by a 30 min treatment with HRP Rabbit/Mouse (Santa Cruz Biotechnology, CA, USA). The section was developed with diaminobenzidene-hydrogen peroxidase substrate, and lightly counterstained with hematoxylin. To calculate microvessel density (MVD), three most vascularised areas of the tumour (‘hot spots’) were selected and mean values obtained by counting vessels. A single microvessel was defined as a discrete cluster of cells positive for CD31 staining, with no requirement for the presence of a lumen. Microvessel counts were performed at X400 (X40 objective lens and X10 ocular lens; 0.74 mm^2^ per field).

### Statistics

One-way ANOVA followed by Tukey's Multiple Comparison Test was performed to determine the significance of differences among groups using GraphPad PRISM version 3.0 software (SanDiego, CA). Differences were considered significant in all experiments at *P*<0.01 (*, significantly different from untreated controls; ^**^, significantly different from Nos and Gem single treatments.

## Results

### Synergistic *in vitro* cytotoxicity of NGC treatment

**Figure 1:**
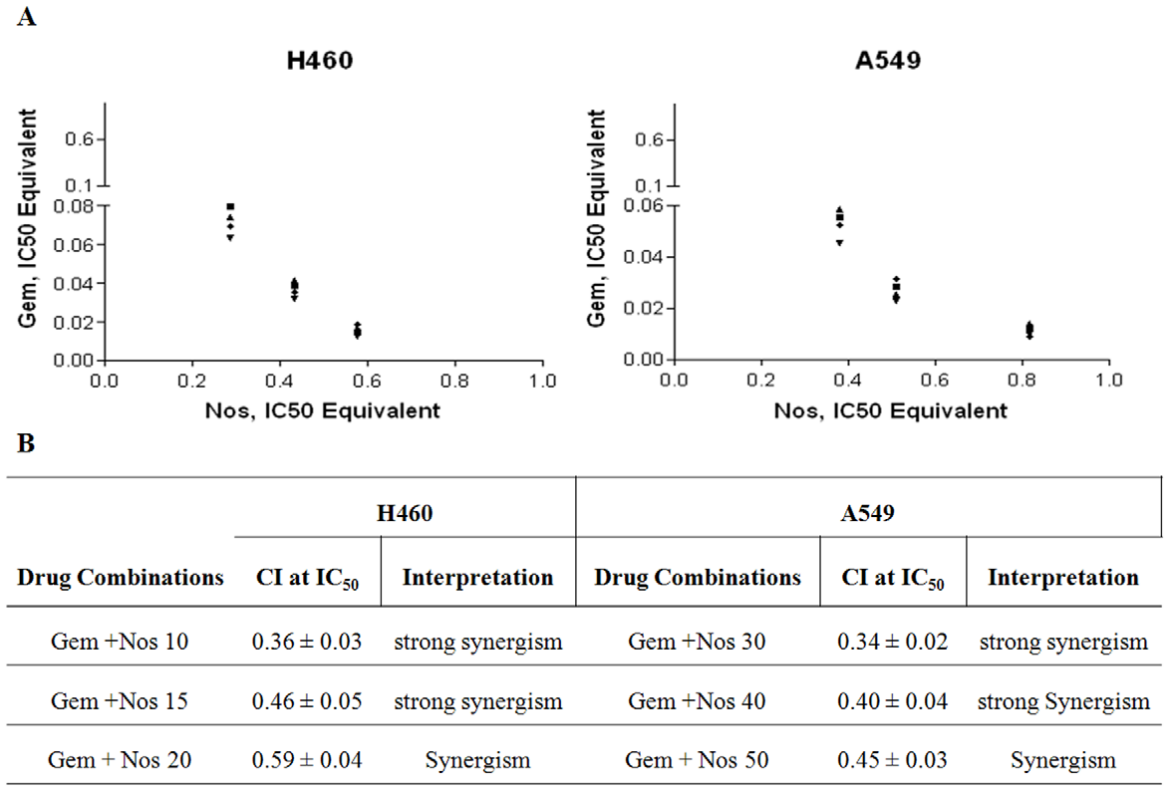
Isobolograms (A) and (B) Combination Index (CI) values of the interaction between Gem with Nos against human lung Cancer cells. Different concentrations of Nos were employed to study the effect on IC50 of Gem. Variable ratios of drug concentrations and mutually non-exclusive equations were used to determine the CI. The CI values represent mean of four experiments. CI >1.3: antagonism; CI 1.1–1.3: moderate antagonism; CI 0.9–1.1: additive effect; CI 0.8–0.9: slight synergism; CI 0.6–0.8: moderate synergism; CI 0.4–0.6: synergism; CI 0.2–0.4: strong synergism.

In vitro cytotoxicity studies with Nos against H460 and A549 cells showed IC_50_ values of 34.7±2.5 µM and 61.25±5.6 µM respectively. Gem showed IC_50_ of 0.7±0.1 µg/ml and 0.6±0.2 µg/ml against H460 and A459 NSCLC cells. The combined effects of Gem and Nos on cell proliferation was evaluated by isobolographic analysis. The CI values ranged from 0.34±0.02 to 0.59±0.04 for 50 percent cell kill suggesting synergistic to strong synergistic behavior between Nos and Gem against both NSCLC cell lines (Fig 1A). Isobolograms for the 50% effect level show that the IC_50_-equivalent concentrations for various Nos/Gem combinations were located below the line of additivity, indicating synergistic activityof Nos and Gem combination (Fig. 1B). The separation of the points in the isobologram was consistent with that of the CI values.

### Antiangiogenic effect- tube formation assay

**Figure 2:**
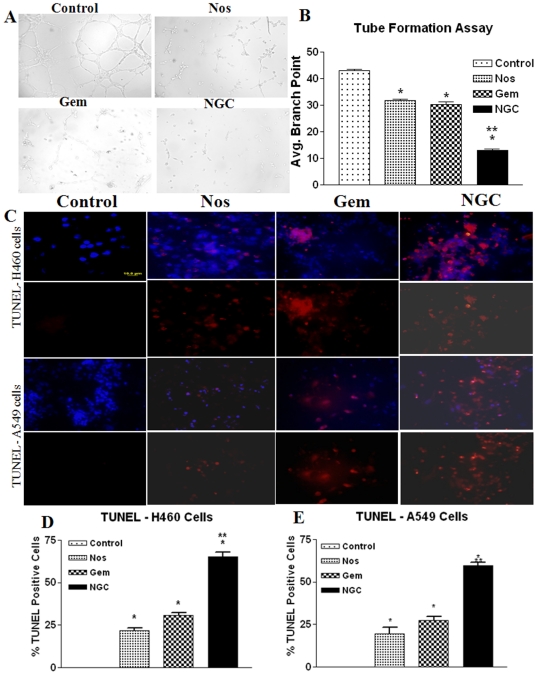
Tube formation assay with HUVEC cells after 6 h (A); quantification of branching points (B); micrographs of cells stained with TUNEL after 72 h (C) and quantitation of apoptotic H460 (D) and A549 cells from TUNEL assay (E). For tube formation assay, HUVEC cells were incubated with Nos (30 µM), Gem (0.4 µg/ml) and NGC on polymerized Matrigel at 37°C. After 6 h, tube formation by endothelial cells was photographed and the capillary tube branch point formation were quantified (n = 3). For TUNEL assay, H460 cells were treated with Gem 0.4 µg/ml, Nos 30 µM, and, NGC and A549 cells were treated with Gem 0.3 µg/ml, Nos 50 µM, and, NGC. Control cells were untreated. Micron bar  = 10 µm. Cells were quantitated by counting 100 cells from 6 random microscopic fields. Data are expressed as mean + SD (n = 6).

Nos (30 µM), Gem (0.3 µg/ml) and 30 µM Nos + 0.3 µg/ml Gem treatment for 6 hr showed poor organizationof HUVEC tube-like structures and a decrease in capillary tube branch point formations (Fig. 2A) compared to control group which showed a rich meshwork of branching anastomosing capillary-like tubules with multicentric junctions (Fig. 2). NGC treatment decreased formulation of average branching point by 68±5 percent compared to 26±3 percent by Nos alone and by 29±4 percent Gem alone (Fig. 2B).

### Induction of apoptosis in H460 and A549 cells

Fig. 2C shows that apoptosis is induced in H460 and A549 cells following treatment with Gem, or Nos, or NGC. NGC treatment led to apoptosis in 59±4 percent of treated H460 cells compared to 27±3 percent and 20±2 percent in Gem and Nos respectively after 72 hr (Fig 2D and 2E). Similarly, NGC treatment of A549 cells led to 67±5.0 percent apoptotic cells compared to 32±2.0 percent and 20±3.0 percent in Gem and Nos respectively (Fig 2D and 2E). All treatments were significantly different from control (* P<0.01). Gem or Nos treatment was significantly different from NGC treatment (**, P<0.001).

### Anti-tumor activity of NGC in H460 xenografts

**Figure 3:**
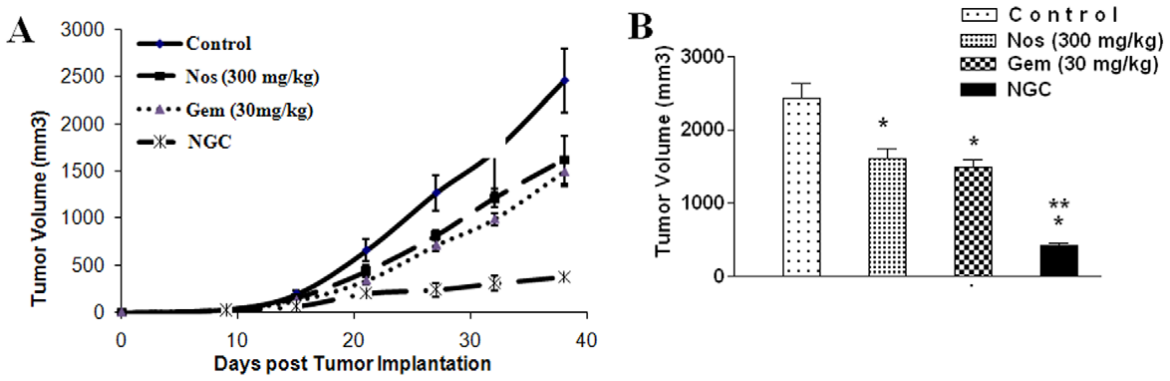
Effects of Nos, Gem and NGC on human H460 lung tumor xenograft model (A) progression profile of tumor growth kinetics and (B) tumor volume measurements on days 38 post-inoculation (tumor volumes, mm3 ± SEM). The mice were treated with Gem 30 mg/kg i.v. bolus, q3d ×7 schedule, Nos 300 mg/kg/day, and NGC. Control group received vehicle only. Data presented are means and SE (n = 8). This experiment was repeated twice.

The results (Fig. 3) show that tumor volume significantly decreased after treatment with Gem, Nos, or NGC compared to control. Tumor volume for the NGC treatment averaged 418.74±55.53 mm^3^ compared with 1605.08±253.29 mm^3^ for Nos treatment or 1498.33±149.38 mm^3^ for Gem treatment (tumor volume ± SE) on day 38 post tumor implantation. Furthermore, we did not observe any weight loss or other signs of toxicity in the mice treated with NGC or Nos or Gem.

### Effects on specificity proteins, angiogenic and apoptotic proteins in H460 xenograft lung tumors

**Figure 4:**
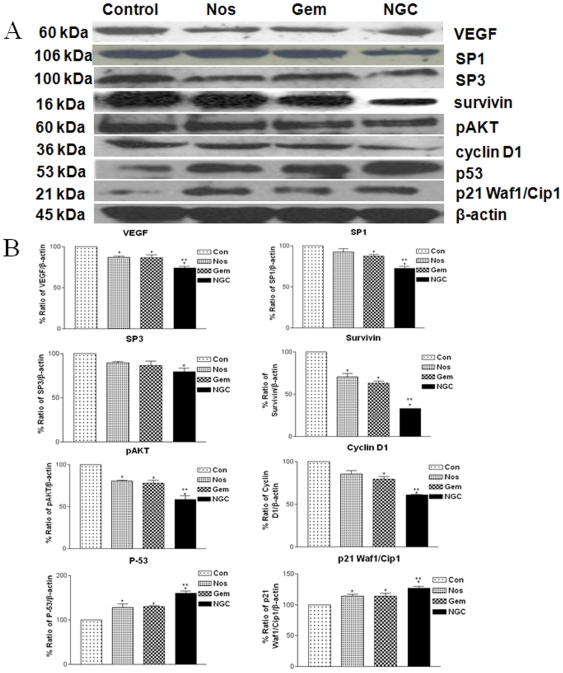
Expression of VEGF, SP1, SP3, pAKT, cyclin D1, p53, p21, and survivin proteins in tumor lysates by western blotting (A) and (B) quantitation of apoptotic protein expression. Lane 1, untreated control tumors; lane 2, oral Nos 300 mg/kg; lane 3, Gem 30 mg/kg i.v. bolus, q3d ×7 schedule ; lane 4; NGC. β-actin protein acts as a loading control. Similar results were observed in triplicate experiments. Protein expression levels (relative to β-actin) were determined. Mean ± SE for three replicate determinations.

**Figure 5:**
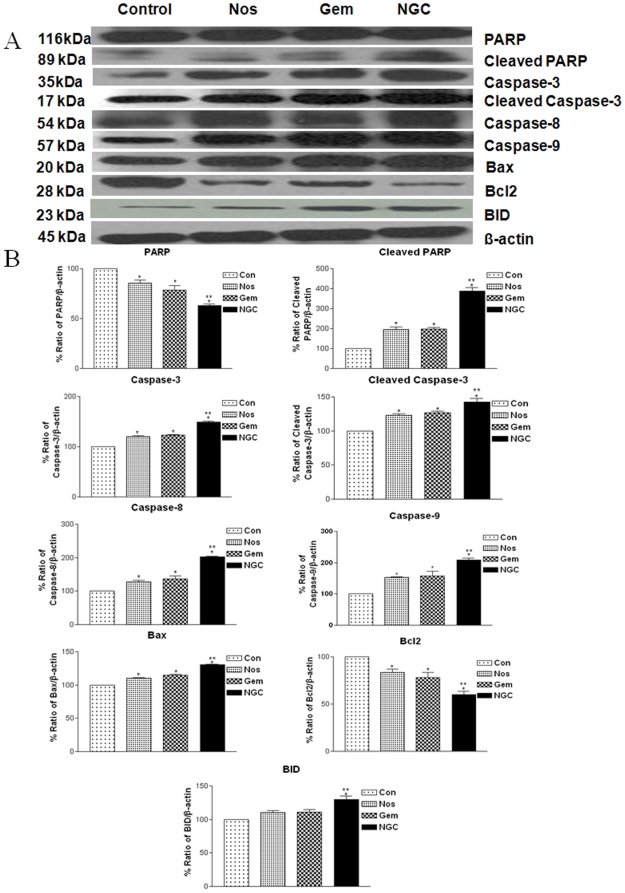
Expression of apoptotic PARP, cleaved PARP, caspase 3, cleaved caspase 3, caspase 8, caspase 9, Bax, Bcl2, and BID proteins in tumors (A) and (B) quantitation of apoptotic protein expression. Lane 1, untreated control tumors; lane 2, oral Nos 300 mg/kg; lane 3, Gem 30 mg/kg i.v. bolus, q3d ×7 schedule ; lane 4; NGC. β-actin protein acts as a loading control. Similar results were observed in replicate experiments. Protein expression levels (relative to β-actin) were determined. Mean ± SE for three replicate determinations.

The NGC and Gem decreased expression of Sp1 and Sp3 in harvested tumors compared to control mice (Fig 4). NGC treatment decreased expression of VEGF protein expression to 0.26-fold compared to 0.12-fold with Nos and 0.13-fold with Gem treatment, respectively of controls in regressed tumors (Fig 4). The expression of survivin protein were significantly decreased by 0.67 fold, 0.29 fold and 0.37 fold with NGC, Gem and Nos treatment compared to control group respectively (Fig 4). The NGC showed significant decrease in expression of pAKT compared to control group. In regressed tumors, the NGC and Gem significantly decreased Cyclin D1 expression to a 0.39, 0.20, and 0.15-fold, respectively of controls (Fig 4). Nos, Gem and NGC treatment significantly increased p53 expression to 1.2, 1.3 and 1.6-fold in regressed tumor samples compared to control respectively. (Fig 4) Expression of p21 was significantly increased to 1.31-fold with NGC treatment compared to 1.15 and 1.13-fold with Nos and Gem treatment respectively (Fig 4). Results illustrated in Fig 5 show that Nos, Gem and NGC treatment showed significant increased expression of cleaved PARP, Bax, BID, caspase 3, cleaved caspase 3, caspase 8 and caspase 9, and decreased expression of PARP and Bcl_2_ compared to control group. The expression of apoptotic and antiapoptotic proteins in NGC treatment was significantly different from single agent treatment groups.

### DNA fragmentation and cleaved caspase-3 expression in tumor tissue

**Figure 6:**
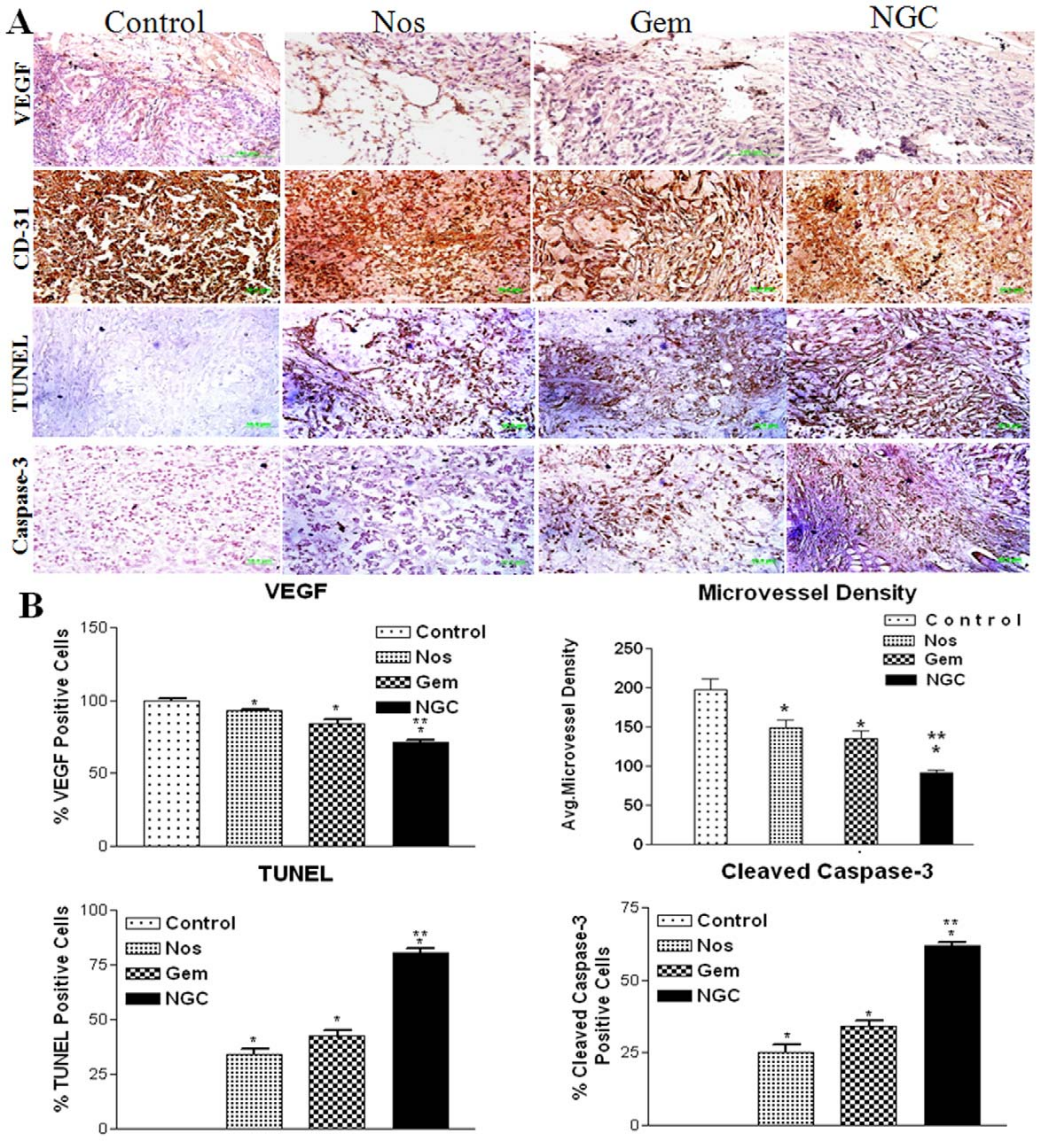
Immunohistochemical staining of H460 tumor tissues for VEGF, CD31, TUNEL assay and cleaved caspase 3 expression (A) and (B) quantitation of quantization of VEGF, assessment of microvessel density, quantitation of TUNEL positive cells and cleaved caspase 3 positive cells. Data are expressed as mean + SD (N = 6).

Single-agent treatment with either Nos or Gem induced DNA fragmentation that was further significantly (** P<0.001) increased by NGC treatment. The NGC treatment led to apoptosis in 80±5.0 (** P<0.01) percent of the tumor cells, whereas Nos and Gem induced apoptosis in 34±3.0 percent and 42±4.0 percent of the tumor cells respectively (Fig. 6). Gem, Nos, and NGC induced cleaved caspase-3 expression in tumors which was significantly different compared to control tumors. NGC, Gem and Nos treatment showed 62±4.0, 34±2.0, and 26± 2.0 percent increased expression of cleaved caspase 3 in tumors tissues respectively compared to control group (Fig. 6).

### Inhibition of angiogenesis and MVD in tumor tissue

The highest expression of VEGF was seen in tumor tissues harvested from untreated mice (Fig. 6). Decreased VEGF staining was observed in tumors treated with NGC (0.28-fold) compared to tumors treated with Gem (0.15-fold) or Nos (0.1-fold) alone. This response was well correlated to down-regulation of VEGF protein observed in tumor lysates from mice treated with the same compounds (Fig. 4). CD31 (+) endothelial cells were identified using IHC technique in harvested tumor tissues and the results are shown in Fig. 6. The staining of microvessels in NGC, Gem and Nos treated groups were significantly decreased to 0.21, 0.08, and 0.06-fold compared to control group. The average microvessel per field in groups treated with Nos, Gem and NGC were found to be 148.7±18.6, 135.3±17.5, and 91.8 6.1 respectively compared to 197.7±22.4 in control group.

## Discussion

Poor clinical outcome of the current chemotherapy necessitates search for newer therapeutic strategies for the treatment of NSCLC [1], [3], [4]. The development of potent nonplatinum based combination chemotherapy with fewer adverse side effects will help to improve the clinical outcome among lung cancer patients [3], [4], [5]. The use of promising anti-microtubular agents has been limited due to drug-resistance, prolonged i.v infusion and associated adverse side effects [15], [16]. Nos is a safer orally active microtubule agent [19], [20], [23] and has exhibited in-vitro and in-vivo antitumor activity against variety of cancers [19], [20], [21], [22], [23]. Our previous studies demonstrated that Nos exhibited anticancer activity in murine H460 xenograft model with no adverse side effects [24]. Gem is one of the most effective agents against lung cancer which inhibits DNA synthesis and ribonucleotide reductase [6], [7]. Thus, it is expected that NGC chemotherapy may exert additive or synergistic anticancer activity and will have lower adverse side effects due to reduced dose requirement for Gem.

The fast growing H460 and slow growing A549 cells [28] were selected to ascertain activity of Nos and Gem combination at sub IC_50_ concentrations using commonly used isobolographic method [28], [29], [30]. In the present investigation, isobolographic analysis of the data showed that Nos enhanced the cytotoxicity of Gem (CI values <0.59) in A549 and H460 human NSCLC cells in a synergistic manner (Fig 1A). The synergistic activity was also observed in the isobologram, where IC_50_-equivalent concentrations for various NGC were located below the line of additivity (Fig 1B). We recently reported that the CI values <1.0 are indicative of synergistic activity between DIM-C-pPhC_6_H_5_and Docetaxel in NSCLC cells [28].

To study the possible mechanism involved in the enhanced cytotoxicity of Gem by Nos, we evaluated HUVEC tube formation and apoptosis of tumor cells [31]. Newcomb et al. reported that Nos (0–150 µM) inhibited *in vitro* tube formation in 2H11 endothelial cells [32]. Similarly, Laquente et al also demonstrated inhibition of proliferation of HUVEC cells by Gem [33] and hence, we have evaluated the antiangiogenic potential of NGC. There has been no study so far to evaluate the effect of GNC on HUVEC tube formation. NGC showed the linear structures of the network were significantly disrupted compared to single agent treatment or controls. NGC exhibited synergistic anti-angiogenic activity by inhibition of tube formation compared to Nos or Gem alone (Fig 2).

Induction of apoptosis is key mechanisms of anticancer agents and the significant induction of apoptosis which was evident from positive TUNEL staining and chromatin condensation in NGC treatment compared to single agent. Similar to our results, combination treatment of Nos (150 mg/kg/day by gavage) and ^60^Co radiation (single fraction - 25 Gy) showed significant (*P*<0.01) increased TUNEL positive GL261 cells compared to single agent treatment [33].

Having established the effectiveness of the NGC treatment *in vitro*, we next evaluated the *in vivo* antitumor efficacy of NGC in H460 xenograft lung tumors in Nu/nu mice. We selected sub-therapeutic doses of Nos (300 mg/kg/dayand Gem (30 mg/kg i.v. bolus, i.v. bolus, q3d ×7 schedule) based on our previous studies which have demonstrated dose dependent anticancer activity (300<450 <550 mg/kg/day) of Nos against H460 xenograft murine model [24]. Our *in vivo* results demonstrate additive behavior of NGC in murine H460 xenograft tumor model (Fig 3A). Previous reported studies demonstrated that anticancer activity of Nos varies with the type and sensitivity of cancer cells [19], [20], [21]. Interestingly, NGC treatment showed non-significant change in weight loss suggesting favorable toxicity profile of Nos and Gem. NGC treatment will be advantageous over conventional Gem + taxane treatment of lung cancer due to improved patient compliance by oral administration of Nos with minimal adverse side effects. Several studies have provided evidence that enhanced tumor growth inhibition can be achieved by combining Gem with other agents such as bortezomib [34], Telomelysin [35], Dihydroartemisinin [36], Paclitaxel [37], and topotecan [38] compared to single agent treatment. The *in vivo* additive activity of NGC treatment may be attributed to poor bioavailability (<30%), shorter plasma half life (<4.5 h) and extensive first-pass metabolism of Nos reducing the availability Nos at the tumor site [39], [40], [41]. Our future studies will focus on improving the bioavailability of Nos to explore its anticancer potential in combination with Gem.

Based on *in vitro* antiangiogenic and apoptotic activity we have evaluated the expression of Sp proteins, cell survival, VEGF and apoptotic proteins. Sp proteins are transcription factors which are overexpressed in many human tumors [42], [43]. Lou et al. demonstrated that transformation of fibroblasts resulted in increase in Sp1 expression by 8 to 18-fold and showed formation of highly malignant tumors in athymic xenograft models [44]. For the time time, we showed that NGC treatment showed significant (p<0.01) decrease in the expression of Sp1 and Sp3 proteins compared to single agent treatment and control group. Previous studies demonstrated that VEGF expression is partially dependent on Sp proteins [45], and there is also evidence that survivin expression is Sp dependent [46]. Gem, Nos and NGC treatment decreased expression of survivin in lung tumors (Fig 4A and 4B). Survivin is a member of the inhibitor of apoptosis family which inhibits caspase activation and acts as a negative regulator [46]. Therefore, the down-regulation of survivin expression results in activation of caspases and thereby induces apoptosis in tumor cells. We also observed that NGC treatment significantly decreased expression of VEGF (Fig 4D) in regressed tumors compared to single agent treatment. Similarly, Papineni et al demonstrated that Tolfenamic acid inhibits esophageal cancer through repression of Sp proteins and several Sp-dependent genes and proteins such as VEGF, survivin, cyclin D1 and Bcl-2 [47]. Nos alone and NGC treatment showed decreased expression of VEGF and survivin, which may be correlated to degradation of Sp1 and Sp3. Furthermore, our IHC results show that NGC treatment decreased VEGF and CD 31 staining in tumor tissues harvested from mice compared to single agent treatment and control (Fig 6A). The tumor regression by NGC was also mediated through decreased expression of VEGF and CD 31 and correlated very well with our VEGF expression western blots results of tumor lysates (Fig. 4D) and inhibition of tube formation (Fig 2). MVD is a commonly used index of tumor angiogenic activity and the average microvessels per field (Fig. 6B) in NGC treated group were significantly (p<0.001) decreased compared to the single agent treated and control group.

Previous studies demonstrated that Nos induces multiple proapoptotic responses that induce apoptosis against variety of tumors [19], [20], [21]. Activation of p53 protein plays a crucial role in the control of tumor cell response to drugs and Bax and p21 are downstream effectors proteins. [48]. Bax, BID and p21, were activated in NGC treatment producing apoptosis in lung tumors cells. In support of our data, Aneja et al demonstrated that anticancer activity of Nos against colon cancer cells was mediated via p53-dependent pathway [48]. Furthermore, the decreased expression of cyclin D1 suggests involvement of ubiquitin/proteosome system in induction of apoptosis by NGC. Ubiquitin/proteosome system regulates various cell cycle regulators and transcription factors such as p53, cyclins, and cyclin-dependent kinase inhibitors. In addition, we also observed that Gem, Nos and NGC decreased cell survival proteins pAKT in lung tumors (Fig 5). Caspases are critical protease mediators of apoptosis triggered by different stimuli [29]. The NGC treatment activated initiator caspases, such as caspase-8 (intrinsic pathway) and caspase-9 (extrinsic pathway) followed by activation of effector caspase-3. Consistent with our results, Schniewind et al demonstrated enhanced cytotoxicity of phenylbutyrate and Gem therapy against KNS62 and Ben NSCLC cells via intrinsic apoptotic pathways [49]. Results of our *in vivo* studies also demonstrate that Gem and Nos alone and NGC treatment induce Bax expression and decrease survival (Bcl_2_) proteins (Fig 5). These results also suggest that apoptosis may be mediated through the mitochondrial pathway via down-regulation of antiapoptotic Bcl_2_ and up-regulation of proapoptotic Bax [24]. DNA fragmentation was significantly induced *in vivo* by NGC treatment compared to Nos or Gem alone thus confirming that apoptosis is one of the underlying mechanism, which is in agreement with our *in vitro* TUNEL assay results and western blots results of tumor lysates (Fig. 5). Our previous studies also demonstrated induction of apoptosis and activation of cleaved caspase 3 following Nos treatment in NSCLC [24]. Our findings suggest that the activation of extrinsic and intrinsic apoptotic pathways plays a critical role in the cytotoxicity of NGC treatment against NSCLC. To gain more insights on the anticancer mechanisms of NGC therapy, other non-apoptotic signaling pathways need to be investigated which are in progress.

In conclusion, our results demonstrate that NGC therapy is highly effective for inhibiting lung tumor growth in a murine lung xenograft model. The antitumorigenic activity of Gem was enhanced by Nos through degradation of Sp proteins, inhibition of angiogenesis, and induction of apoptosis via intrinsic and extrinsic pathways in lung tumors. Thus the use of NGC therapy could be an innovative and promising therapeutic strategy for the treatment for lung cancer and possibly will have fewer adverse side effects compared to currently available platinum based chemotherapy.
